# Library of Disordered Patterns in 3D Protein Structures

**DOI:** 10.1371/journal.pcbi.1000958

**Published:** 2010-10-14

**Authors:** Michail Yu. Lobanov, Eugeniya I. Furletova, Natalya S. Bogatyreva, Michail A. Roytberg, Oxana V. Galzitskaya

**Affiliations:** 1 Institute of Protein Research, Russian Academy of Sciences, Pushchino, Russia; 2 Institute of Mathematical Problems of Biology, Russian Academy of Sciences, Pushchino, Russia; 3 Pushchino University, Pushchino, Russia; Institute of Enzymology, Biological Research Center, Hungarian Academy of Sciences, Hungary

## Abstract

Intrinsically disordered regions serve as molecular recognition elements, which play an important role in the control of many cellular processes and signaling pathways. It is useful to be able to predict positions of disordered regions in protein chains. The statistical analysis of disordered residues was done considering 34,464 unique protein chains taken from the PDB database. In this database, 4.95% of residues are disordered (i.e. invisible in X-ray structures). The statistics were obtained separately for the *N*- and *C*-termini as well as for the central part of the protein chain. It has been shown that frequencies of occurrence of disordered residues of 20 types at the termini of protein chains differ from the ones in the middle part of the protein chain. Our systematic analysis of disordered regions in PDB revealed 109 disordered patterns of different lengths. Each of them has disordered occurrences in at least five protein chains with identity less than 20%. The vast majority of all occurrences of each disordered pattern are disordered. This allows one to use the library of disordered patterns for predicting the status of a residue of a given protein to be ordered or disordered. We analyzed the occurrence of the selected patterns in three eukaryotic and three bacterial proteomes.

## Author Summary

In this work we have studied the statistics of disordered amino acid residues in the Protein Data Bank (PDB). It has turned out that 42% of disordered residues are near the *N*-terminus of proteins, 30% are near the *C*-terminus, and the remaining 28% are in the middle part of protein chains. It has been shown that the relative frequencies of occurrence of disordered residues at the termini of protein chains differ from the ones in the middle part of protein chains. We have compiled the first and largest database of disordered patterns from the PDB (version from 28 June 2010). Pattern analysis of the distribution of disordered regions in the PDB demonstrated that the disordered patterns are length and position dependent. The results of these analyses help to further our understanding of the physicochemical and structural determinants of intrinsically disordered regions that serve as molecular recognition elements. As expected, the occurrence of patterns in the bacterial proteomes is considerably less than in the eukaryotic proteomes.

## Introduction

Prediction of protein structure and function is one of the general directions in structural genomics. Of special interest is prediction of the so-called disordered regions of protein chain (regions having no fixed spatial structure in the native state). Such disordered regions often play an important functional role ([1]–[6]). It should be emphasized that one type of disordered regions are structured only when they bind (bound) to other molecules [3], , or under changing the conditions of biochemical medium [9], [10], but the other kinds of disordered regions are always disordered and never become structured. Disordered regions of protein chains often cause complications upon expression, purification and crystallization of such proteins.

At present, more than 500 proteins with disordered regions are described in the Disprot database [11]. These proteins and domains are either entirely unstructured in the native state (the so-called natively-unfolded proteins) or have lengthy disordered regions. At that functionally important protein regions in such proteins are outside of globular domains, i.e. just in the disordered regions [9], [11].

Since disordered regions of the protein chain play an important role in the protein functioning, much attention is being paid to their examination and prediction [12], [13]. Indeed it has been shown that disordered proteins have certain properties which distinguish them from proteins with well-defined structures [14]. Abundance of intrinsic disorder in PDB was discussed in a recent study [14]. Typically, disordered regions have a low aromatic content and high net charge as well as low sequence complexity and high flexibility [15]–[19].

Prediction methods aim to identify disordered regions through the analysis of amino acid sequences using mainly the physico-chemical properties of the amino acids [20]–[29] or evolutionary conservation [30]–[33].

It can be suggested that if one and the same pattern corresponds to disordered regions in the protein structures then it is highly probable that such a pattern will be disordered in other proteins.. Search for disordered patterns is an important task for prediction of disordered regions and search for the functioning of the considered motifs. The identification of essential features within protein domains can greatly facilitate their functional characterization. There are well established databases on protein motif or domain information, such as PROSITE, InterPro and Pfam [34]–[36].

Creation of a library of disordered patterns is one of the primary tasks in this respect. There is no information about such a library. Until now we have known the PEST motif (*i.e.*, regions locally enriched in proline, glutamic acid, serine, and threonine and, to a lesser extent in aspartic acid) which in most cases is a degradation motif [37] and the RGD motif which can be found in extracellular matrix proteins such as fibronectin, fibrinogen, prothrombin, tenascin, thrombospondin, vitronectin, and *etc.* [38],[39]. The exposed RGD motif constitutes a major recognition site for integrin binding [40].

In this work we have been interested in stretches of disordered residues (a minimal length is six residues). As a rule such stretches are short loops inside globular domains and present only one type of disorder, because disordered proteins range from molten globules to chains having no structural preferences whatsoever (in terms of flexibility) and from 2–3 residues to several hundreds or even thousands of residues (in terms of length) [3], [11]–[13]. We have analyzed disordered regions and have created a library of disordered motifs and their positions in protein chains from the entire Protein Databank (PDB version from 28 June 2010) [41]. Taking into account the consideration of the library of disordered patterns will help in improving accuracies of predictions for residues to be structured or unstructured inside the given region. Moreover, our new statistics on the occurrence of unstructured residues will be useful for those who are dealing with prediction of the status of residues to be ordered or disordered.

Combining the motif discovery and disorder protein segment identification in the PDB is a new and promising approach for further studying and understanding the functional role of the obtained patterns in different proteomes. The question about specificity of these patterns is more important for biological functioning. We have analyzed the occurrence of the obtained patterns in some eukaryotic proteomes (humans, the fruit fly, and the nematode worm proteomes) and in some bacterial proteomes (*E.coli*, *Lactococcus lactis*, and *Mycobacterium tuberculosis*).

## Materials and Methods

### Preprocessing of data

We have considered all protein structures determined by X-ray analysis with a resolution better than 3 Å, published in the PDB (version from June 28, 2010); the structures contain 116 997 protein chains. Approximately 4.5% of their residues (see below) are disordered, i.e., are not resolved by X-ray analysis. To identify such residues, we have compared (for each protein chain) the records SEQRES and the records ATOM in the corresponding PDB-file. Residues which are present in the record SEQRES, but their coordinates are absent in the record ATOM (namely, the coordinates of the C_α_-atom are absent in the record ATOM), are considered as unstructured ones.

These 116 997 chains can be divided into 34 464 classes, the chains from the same class have the same amino acid sequences, the sequences of chains from the different classes are different i.e. differ at least at one position. In total these 34 464 different sequences contain 9 085 893 residues.

**Figure 1:**
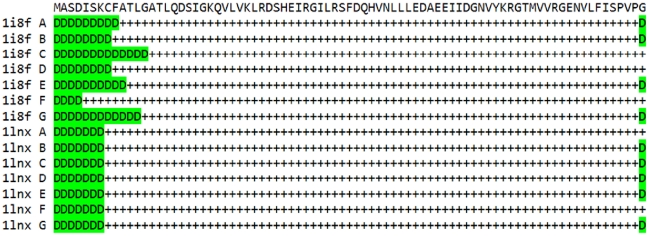
Illustration of definition of disordered fraction. The given protein chain occurs in two PDB files: 1i8f and 1lnx. The *C*-terminal glycine is disordered in nine out of 14 cases. Therefore, for glycine the weight to be disordered is 9/14 and to be ordered is 5/14, correspondingly. For this example, there are 8.7 average disordered residues.

We have created the Disordered Residues Data Base (DRDB), its elements are 34 464 sequences from the PDB (version from June 28, 2010). For the *i*-th residue of a given sequence *S* we have calculated fraction DF(*i*, *S*) of the corresponding PDB chains where the residue is disordered. Figure 1 illustrates the definition of DF(*i*, *S*). It shows 14 chains with the same sequence (given at the top). ‘D’ means that the corresponding residue is disordered, while ‘+’ means that the residue is resolved. For example, *C*-terminal glycine (position 81) is not determined in nine cases from 14 (chains A, B, E, etc). Therefore, the weight DF(81, S) for glycine-81 and the sequence S shown in Figure 1 is 9/14. The database DRDB stores values DF(*i*, *S*) for all residues of all sequences. 8 592 356 residues (94.57%) are perfectly ordered (DF(*i*, *S*) = 0) and 376 644 (4.15%) are perfectly disordered (DF(*i*, *S*) = 1); the intermediate cases comprise 1.29% of all residues. Below it is stated that residue *i* of sequence *S* is disordered if DF(*i*, *S*)≥0.5. The total number of such residues is 449 584 which makes 4.95%. The average value 
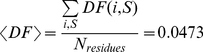
 over all residues.

Below we consider only elements of the DRDB, thus words “chain” and “sequence” are synonyms, each of them denotes an element of the DRDB.

### Disordered regions and estimation of their quality

Our goal is to create a database of disordered patterns, i.e. amino acid sequences that are likely to be found in disordered parts of protein chains. Let *P* be a protein chain and *A* be a pattern of length *L*. The database was compiled using a two-stage procedure. At the first stage we created a list of candidate patterns. Then the desired disordered patterns were selected from the candidate list.

We say that pattern *A* matches chain *P* at position *s* if

1. there are at most *L/5* positions *r* in which

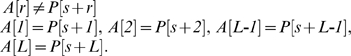

Protein *P* has an occurrence of pattern *A* if *A* matches *P* at position *s*.

Let *TP(A)* be the number of disordered residues in all occurrences of pattern *A* (“true positives”) and *TN(A)* be the number of all ordered residues that do not belong to any occurrence of *A* (“true negatives”). To estimate the “disorder quality” of region *A*, we use the following measures [42]:
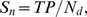
(1)

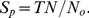
(2)

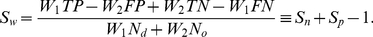
(3)


Here *S_n_* is the sensitivity, *S_p_* is the specificity, *N_d_* is the total number of disordered residues in the DRDB, and *N_o_* is the total number of ordered residues in the DRDB. Thus, sensitivity is a fraction of correctly predicted unstructured residues, and specificity is a fraction of correctly predicted structured residues [42]. *S_w_* is an integral measure used in the CASP competition (“Community Wide Experiment on the Critical Assessment of Techniques for Protein Structure Prediction” is a competition devoted to the evaluation of the quality of prediction of 3D protein structure) in the category devoted to the evaluation of the quality of prediction of unstructured residues [43], [44]. Note that in [43] the formula for *S_w_* is given as follows:
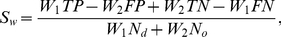
(4)where *FP* (“false positives”) is the number of false positive predictions (the number of residues predicted as unstructured although these residues are in fact structured), *FN* (“false negatives”) is the number of false negative predictions: the number of residues predicted as structured although these residues are in fact unstructured, and 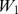 and 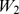 are coefficients calculated as follows: 
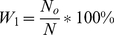
, 
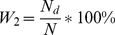
 (*N* = *N_d_*+*N_o_* is the total number of amino acid residues).

However, the definitions are equivalent. As seen, the equation for calculation of *S_w_* can be rewritten using a smaller number of symbols than that in [43]. Substituting equations instead of *W_1_* and *W_2_*, we obtain:
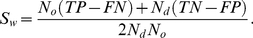
(5)Taking into account, that 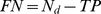, and 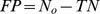, we have:
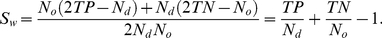
(6)Or, using the definitions for sensitivity and specificity given above, we obtain:
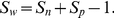
(7)


### Compilation of database of disordered patterns in globular protein

We have designed the database using a two-stage procedure. At the first stage we form the list of candidate patterns. Then the desired disordered patterns are selected from the candidate list.

Fragment *A = P_j_[k, l]* of chain *P_j_* is considered as a candidate disordered pattern if it meets the following conditions:

C1) all residues of the fragment are disordered;
C2) the length of a fragment is at least 6;
C3) fragment *A* has occurrences in at least 5 other unique chains from DRDB.

We select disordered patterns from the candidate list using the following iterative greedy procedure. Let *C* be a chain, and *C[k, k+l-1]* be an occurrence of pattern *A*. The occurrence is terminal if it belongs to the first 40 residues (“*N*-terminal”) or last 40 residues (“*C*-terminal”) of the chain. The other occurrences are called internal ones.

Let DD be a set of candidate patterns. Residue *r* of chain *C* is called the *DD-residue* if

- it belongs to the occurrence of the pattern from *DD*, or
- *r* lies between the *N*-terminus and the *N*-terminal occurrence of the pattern from DD, or
- *r* lies between the *C*-terminus and the *C*-terminal occurrence of the pattern from DD.

Let *TP(DD)* be the sum of disorder coefficients DF for all DD-residues; *TN*(*DD*) be the sum of 1-DF for all non DD-residues.

Let candidate patterns D_1_, …. D_k_ be already included in the database; *B* = {D_1_, …. D_k_}. Let *T* be a candidate pattern that does not belong to *B*. We denote:
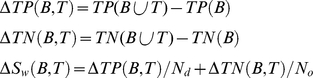



The next candidate to be added to the database is candidate *T* having the maximal value Δ*S_w_(B, T)* among the candidates meeting the following conditions:

S1) 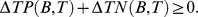

S2) 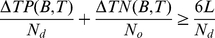 or 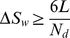, where *L* is the size of the pattern.

If there are no patterns meeting the conditions S1 and S2 then the procedure stops.

There are 856005 regions meeting conditions C1 and C2. The number of regions that also meet condition C3 and conditions S1, S2 with empty set B is 40 411 (here Δ*TP*(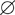, *T*) = *TP(T)* and Δ*TN*(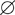, *T*) = *TN(T)*). As a result of the iterative algorithm 426 patterns were identified. The given procedure allows us to choose the minimal set of patterns in such a way as to these patterns included the maximal number of disordered residues.

At last, we are interested in the patterns which will occur in nonhomologous proteins. Therefore, we define a group of proteins as a set of proteins having the same disordered pattern and with identity between protein chains exceeding 20%. Identity between proteins from different groups is less than 20%, correspondingly. We decided to consider only the patterns which appear at least in six groups (with 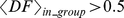). The number of disordered residues in the pattern for proteins from the above mentioned six groups (or larger number of groups) is more than a half. Considering such conditions we want to guarantee that our patterns will occur in nonhomologous proteins. After such a procedure we obtained 109 patterns (see Dataset S1). This procedure allows us to eliminate the patterns occurring only in homologous proteins. Probably, the unstructured conformation of the above patterns connected with the three dimensional structure of these homologous proteins (for example the linker between two domains, a full disordered domain, and etc.).

### Statistical significance of patterns

We have studied statistical significance of the selected patterns from two points of view. First, we have been interested whether the patterns are overrepresented in the database (see #1) and second, whether the disordered fragments are overrepresented among the occurrences of each pattern (see #3).

#### #1 Number of occurrences

To evaluate the statistical significance of the observed number of occurrences of pattern *A* we have calculated the probability *p*(*A*, *N*) that pattern *A* matches a random sequence of length *N*. Here *N* is an average length of a protein (264 in our case). The probability distribution on protein sequences is assumed to be Bernoullian, the probabilities of amino acids are taken from our PDB data set.

The statistical significance of pattern *A* is estimated with the Z-score
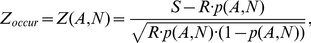
(8)where

*S* is the number of sequences containing at least one occurrence of pattern *A*.
*R* = 34 464 is the number of proteins in the database;
*N* = 264 is the average length of proteins in the database.

To compute the *p*(*A*, *N*) value, we have used two different approaches depending on length *L* of pattern *A*. For patterns of length 15 and less we have computed the probability using algorithm SufPref [45]. Unfortunately, the algorithm cannot process efficiently a long pattern due to the large number of words having at most 20% mismatches with the pattern. To overcome this problem for patterns with the length greater than 15, we have calculated an upper bound
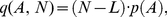
(9)where *L* is the length of pattern *A*; *p(A)* is the probability that *A* matches a random sequence of length *L* (see Dataset S1).

This formula means that we ignore possible overlapping occurrences.

Computer experiments with short words show that the normalized difference
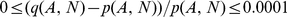
(10)if 10<*L*≤15.

The details of computation of *p(A)* are given below (see #2).

The distribution of Z-scores can be approximated by a normal distribution. We think that a pattern is significant if its *Z*-score exceeds a proper *q*-quantile. We have considered 99-quantile and 95-quantile. For a normal distribution 99-quantile and 95-quantile are equal to 2.33 and 1.65, respectively.

#### #2 Approximate calculation of *p(A)*

To describe the computation of *p(A)*, we need an additional notation. Consider pattern *A* of length *L*. If *A* matches word *V* then *A* and *V* have the same two first and two last letters, therefore all mismatches are possible only at positions *{3, …, L-2}*. Consider partition *{g_1_,..,g_s_}* of positions *{3, …, L-2}* into groups defined as follows: positions *k*, *j* belong to the same group if they are occupied with the same amino acid. Let *d* be a number of mismatches; *0*≤*d*≤*r* where *r = L/5* is the maximal allowed number of allowed mismatches.

*Definition*. A vector *T* = {*d_1_*,…, *d_s_*} is *a mismatch partition vector* for pattern *A* and *d* mismatches if

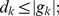

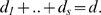

Informally speaking, *d_k_* is the number of mismatches within the positions of group *g_k_*.

*Example*. Consider pattern SHHHHHHSQDP of length *L = 11*. After removal of two first and two last letters we obtain the word HHHHHSQ of length 7 (the word occupies positions from 3 to *L-2* = 9 of the initial pattern). The allowed number of mismatches is *r* = [11/5] = 2. According to the amino acid probabilities the set of positions {3, 4, …, 9} can be divided into three groups: *g_1_* = {3, 4, 5, 6, 7} (corresponds to H); *g_2_* = {6} (corresponds to S); *g_3_* = {7} (corresponds to Q). Let *d = r = 2*. Then the following vectors *T* are possible:
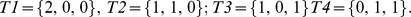



The sum of the elements for each of the vectors is equal to 2, i.e. to the total number of mismatches. Vector *T1* = {2, 0, 0} corresponds to the words where both mismatches are mismatches of H (in other words belong to group *g_1_*), e.g. as in SHAHHAHSQDP. Vector *T2* = {1, 1, 0} corresponds to the words where one mismatch is a mismatch of H and the other is a mismatch of S, e.g. as in SHHHHHATQDP.

For the case *d = 1* we have only 3 mismatch partition vectors:

{1, 0, 0}, {0, 1, 0} and {0, 0, 1}. *End of example*.

*Definition*. Let *d* be a number of mismatches and *T* = {*d_1_*,…, *d_s_*} be a mismatch partition vector. Then *F*(*A*, *d*, *T*) is the set of all words *W* of length *L-4* such that

1. *W* differs from *A* exactly in the *d* positions;
2. Exactly the *d_i_* mismatches are situated in positions from group *g_i_*.

*Proposition*. Let *d* be a number of mismatches and *T* = {*d_1_*,…, *d_s_*} be a mismatch partition vector and *M* be the number of all mismatch partition vectors for pattern *A* and the number of mismatches *d*. Let *p_i_* be the frequency of amino acid at the *i*-th position of pattern *A*.

Then

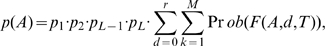
(11)

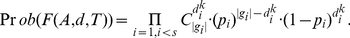
(12)

*Proof*. Follows from elementary combinatorial calculations and is omitted.

*Remark*. Note that number *M* can be calculated by the formula, where 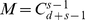 and *s* is the number of groups. In the above example *s = 3*; value *M = 4* for *d = 2* and *M = 3* for *d = 1* (*d* is the number of mismatches).

#### #3 Significance of disordered occurrences

We say that residue *r* of chain *C* is disordered if it is disordered in the majority of representatives of *C* in the considered set of structures (see section Materials and Methods, preprocessing of the data). Fragment *F* of chain *C* from the DRDB database (see section Materials and Methods, preprocessing of the data) is disordered if at least half of its residues are marked as disordered. To estimate the significance of the number of disordered occurrences of pattern *P* we have implemented the following procedure. First, the list of all occurrences of pattern *P* was compiled. Second, we excluded from the list disordered occurrences having intersection with

- an ordered occurrence of the pattern;
- another disordered occurrence of the pattern that is closer to the *N*-terminus than the occurrence under consideration.

Among the remaining *N*(A) fragments we consider the number of disordered fragments *N_d_*(A). The significance of disordered occurrences is estimated with the *Z*-score:
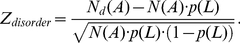
(13)


Here *L* is the length of pattern *P*; *p(L)* is the fraction of disordered fragments within the set of all fragments of length *L* in the database.

## Results/Discussion

### Statistical analysis of distribution of disordered amino acid residues in protein chains

**Figure 2:**
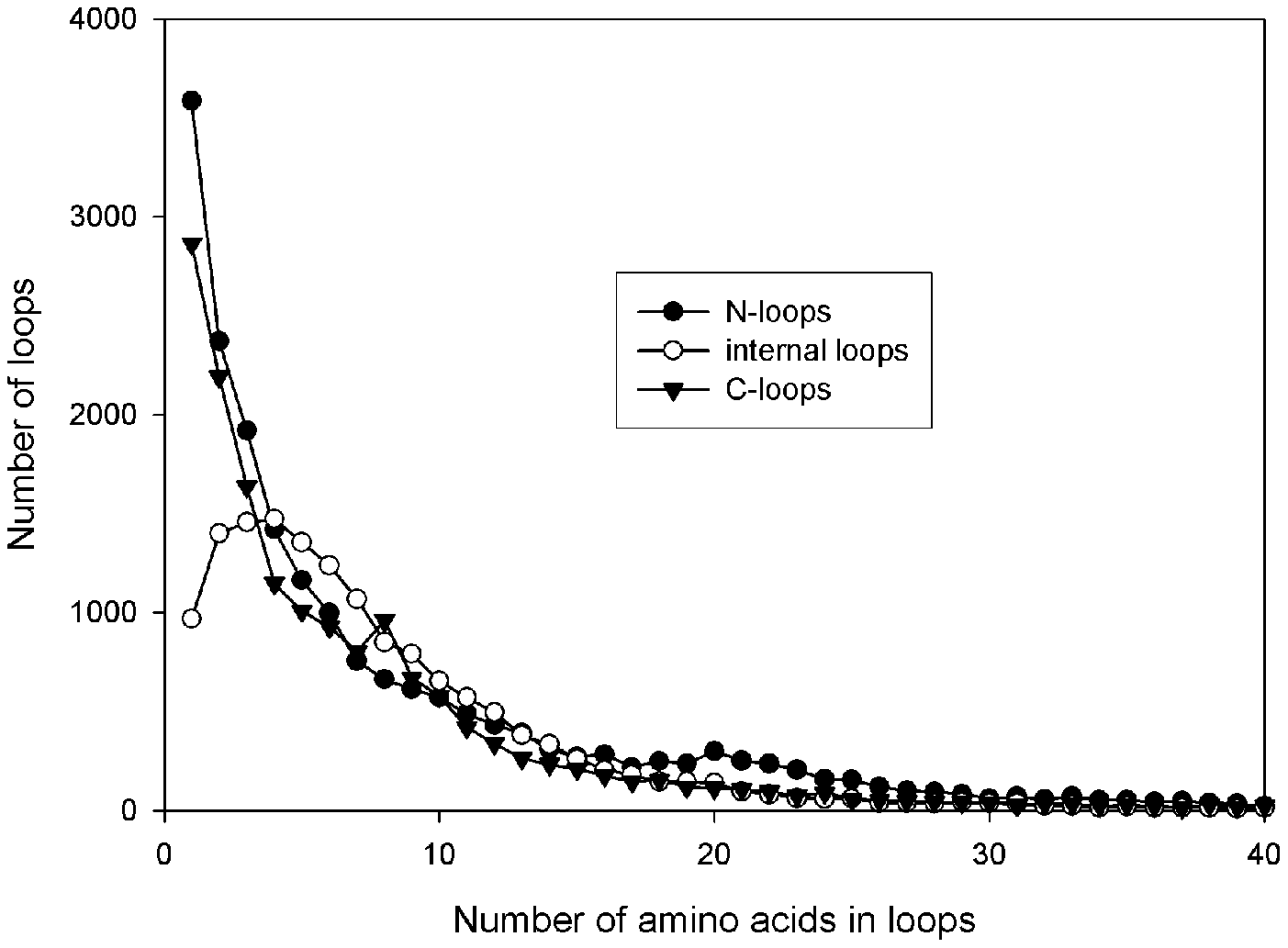
Length distribution of disordered regions in protein chains from the DRDB.

We have analyzed the distribution of disordered residues in the obtained database DRDB, see Materials and Methods. The statistics of the occurrence of disordered regions of different lengths has been calculated. The *N*-terminal disordered regions and the *C*-terminal ones, and internal disordered loops (disordered regions at the both termini of which there are ordered regions) have been considered separately. The distribution of disordered regions by their lengths is shown in Figure 2. As seen, the disordered regions in one residue occur more frequently at the *N*- and *C*-termini of proteins. Disordered regions in four residues occur most frequently in the middle part of the protein chain.

The statistics of distribution of disordered residues in protein chains is given in Table 1. It is interesting that 72% of all disordered amino acid residues are near the termini of protein chains (at a distance less than 40 residues from the *N*- or *C*-terminus of the protein chain), these terminal regions including only 28% of amino acid residues of protein molecules. Therefore for further studying the occurrence of disordered residues we considered separately the terminal regions and the middle part of the protein chain (all the other residues).

**Table 1:** Distribution of disordered amino acid residues in protein structures from the DRDB.

	Fraction of all residues	Fraction of disordered residues
**Terminal parts**	**30%**	**72%**
40 residues near the *N*-terminus	15%	42%
40 residues near the *C*-terminus	15%	30%
**Middle part (all the other residues)**	**70%**	**28%**

**Figure 3:**
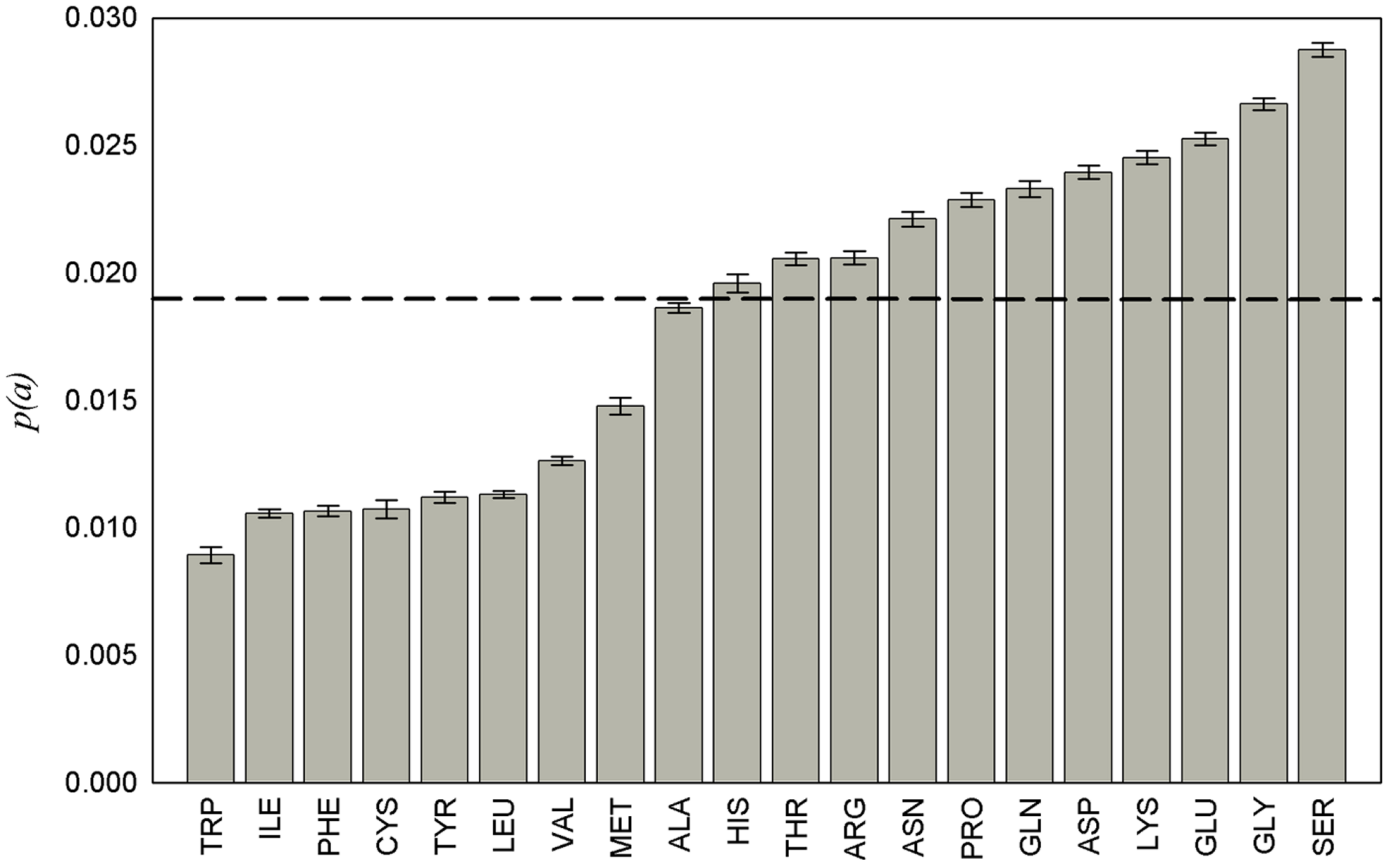
Fraction of disordered amino acid residues for each of the 20 types in the middle part of a protein chain. The dashed line shows the total fraction of disordered residues in the middle part of the protein chain.

The fraction of disordered amino acid residues for each of the 20 types in the middle part of protein chain is presented in Figure 3. As seen from the presented histogram, the fraction of disordered residues in the middle part of a protein chain varies from 0.009 (for tryptophan) to 0.029 (for serine). As should be expected, the fraction of disordered amino acid residues is lower for hydrophobic residues and higher for the hydrophilic ones. It is interesting that serine is more often disordered than any other type of amino acid residues (including glycine and proline which, at least one of them, are usually chosen [20], [25], [46] as residues with a higher “predisposition” to be in disordered regions). The errors indicated on the histogram show that the difference is reliable. The probability to be disordered for the given amino acid residue is calculated according to equation: 
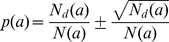
, where *N_d_(a)* is the number of disordered residues, *N(a)* is the number of the given amino acid residue in our database. We can consider *N_d_(a)* as the value which is distributed on the binomial law with probability *p(a)* and *N(a)* is the number of trials. Then, the dispersion is equal to 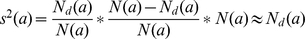.

The probabilities of the occurrence of disordered residues in the middle part of a protein chain and through whole proteins are given in Table 2. As can be seen from the table, serine has a high probability to be disordered both in the middle part of a protein chain and in the whole protein. On the contrary, the probability of methionine to be disordered in the middle part of a protein chain is only a little higher than that of hydrophobic residues, whereas in the whole protein methionine has the highest probability, as compared to the other 20 types, to be disordered (0.093).

**Table 2:** Fraction of disordered amino acid residues for each of the 20 types in the termini, in the middle part of protein chains, and in the whole proteins.

a.a.	TRP	ILE	PHE	CYS	TYR	LEU	VAL	MET	ALA	HIS
*N*-40	0.032	0.054	0.061	0.044	0.055	0.077	0.069	0.351	0.134	0.427
*C*-40	0.029	0.046	0.045	0.047	0.038	0.063	0.054	0.065	0.090	0.376
middle	0.009	0.011	0.011	0.011	0.011	0.011	0.013	0.015	0.019	0.020
whole	0.015	0.022	0.022	0.022	0.021	0.028	0.027	0.093	0.046	0.166

a.a.	THR	ARG	ASN	PRO	GLN	ASP	LYS	GLU	GLY	SER
*N-*40	0.110	0.108	0.115	0.143	0.121	0.108	0.105	0.112	0.167	0.219
*C*-40	0.079	0.087	0.092	0.107	0.100	0.097	0.104	0.117	0.114	0.123
middle	0.021	0.021	0.022	0.023	0.023	0.024	0.025	0.025	0.027	0.029
whole	0.043	0.044	0.046	0.053	0.051	0.046	0.050	0.054	0.060	0.075

### Construction of a library of disordered patterns

Following the procedure described in the Materials and Methods section, we obtained a library of disordered patterns. To our knowledge this is currently the first and the largest database of disordered patterns constructed from the PDB. The dataset includes 109 patterns. The distribution of the patterns on lengths demonstrates that the patterns occur more often as short fragments (75 from 109 are patterns of 6 amino acid residues). The largest pattern consists of 22 amino acid residues. We suggest that these patterns will be disordered when they appear in new protein chains because more than half of residues in these patterns are disordered (see the following section). The shorter the considered pattern the larger the number of groups of proteins with identity lower than 20% among proteins from different groups we obtained where such a pattern appears (see Dataset S1).

Such a rather small size of patterns can be explained by the methodology of extraction of patterns from the DRDB, since we consider the residues situated more closely to the end than the pattern as disordered residues (see section Compilation of database of disordered patterns in globular protein in Materials and Methods).

Some patterns appear in protein together with other patterns. We say that two patterns are “correlated” if there are at least 4 proteins containing both patterns and the identity between the proteins is no more than 20%. The cutoff 4 (4 = 6/2+1) corresponds to the cutoff 6 (the number of groups with identity between proteins from different groups less than 20%, see Materials and Methods, Compilation of database of disordered patterns in globular protein). We found 363 pairs of correlated patterns (that is 6.2% of all possible pairs of 109 patterns). The list of the pairs of correlated patterns is given in the Dataset S2.

**Figure 4:**
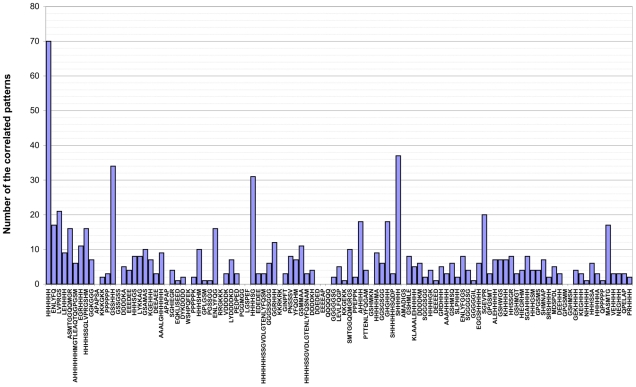
Number of the correlated patterns with the considered pattern in the DRDB. Two patterns are correlated if there are at least 4 proteins containing both patterns and the identity between the proteins is no more than 20%.

In particular for each pair we give the average distance between the pattern occurrences. One can see that approximately in half pairs <d> <0 that corresponds to the intersecting patterns. For example, HHHHHH appears together with 70 other patterns and intersects with 36 of 70 patterns (see Figure 4 and Dataset S2). Pattern LVPRGS occurs 627 times of 828 together with pattern HHHHHH (GSS**HHHHHH**SSG**LVPRGS**). On the other hand, pattern HHHHHH intersects with pattern LEHHHH. We consider that many of the 70 patterns including poly H fragments are artificial parts of proteins which have been added for better purification of proteins. However, poly H fragments appear often in eukaryotic proteomes and likely it has a functional role in comparison of their role in PDB. Moreover, the other 39 patterns can be considered as biologically important; so we found several appearances of these patterns in human proteome (see Table 3 and Dataset S1). The question about specificity of these patterns is more important for biological function and will be considered below. A detailed analysis of the patterns correlation is a subject of future work.

**Table 3:** Occurrence of patterns in the eukaryotic proteomes.

Pattern	Number of groups, identity inside group >20%	Fraction of disordered residues in the patterns from the DRDB	Probability of occurrence of the patterns in protein	Occurrence in the human proteome/in the DRDB	Occurrence in the fruit fly proteome/in the DRDB	Occurrence in the nematode worm proteome/in the DRDB
PPPPPP	15	0.70	0.00017	703/32	304/32	247/32
QQQQQQ	11	0.66	0.00004	331/17	869/17	249/17
EEEDEE	55	0.65	0.00015	242/55	42/55	54/55
QPPPPP	9	0.74	0.00013	163/16	66/16	32/16
APAPAP	17	0.51	0.00067	121/30	44/30	34/30
HHHHHH	1227	0.93	0.00002	99/5423	133/5423	57/5423
EDEDEE	23	0.64	0.00014	97/29	27/29	42/29
DEEEED	12	0.68	0.00014	83/16	26/16	39/16
GGGGGSG	17	0.65	0.00028	78/29	80/29	8/29
GSSGSS	66	0.68	0.00120	67/93	35/93	19/93
PPPPPK	18	0.81	0.00027	62/31	24/31	32/31
DDEDED	14	0.64	0.00013	53/16	31/16	26/16
SGGGGSG	10	0.82	0.00022	31/29	19/29	2/29
KKKGKK	26	0.55	0.00181	27/56	8/56	13/56
EEEEAP	12	0.66	0.00028	26/21	6/21	9/21
KKRKRK	12	0.54	0.00067	25/19	6/19	7/19
SGGGSGG	12	0.68	0.00024	20/17	17/17	5/17
SHHHHH	558	0.98	0.00005	19/1566	27/1566	12/1566
GGSGSGG	17	0.77	0.00027	14/50	23/50	6/50
NHHHHH	19	0.83	0.00003	10/25	14/25	8/25

### Statistical significance of the obtained patterns

We have studied the statistical significance of the selected patterns from two points of view. First, we have been interested whether the disordered fragments are overrepresented among the occurrences of each pattern, and, second, whether the patterns are overrepresented in the database. The features are described with the proper Z-scores (see Materials and Methods), called *Z_disorder_* and *Z_occur_* respectively.

All 109 patterns have *Z_disorder_>9* that corresponds to P-value 10^−19^, which is in good agreement with the procedure of the disordered patterns determination. What is more surprising, the majority of the patterns are overrepresented in the database (89 of 109 have *Z_occur_>5* which corresponds to P-value 3·10^−7^). For a normal distribution 99-quantile and 95-quantile are equal to 2.33 and 1.65, respectively. There are only 7 and 3 patterns with validation less than 2.33 and 1.65, respectively.

### Occurrence of patterns in three eukaryotic and three bacterial proteomes

After creating the library of disordered patterns taken from the PDB, another interesting question arises: how often the obtained patterns will occur in some proteomes. Since the eukaryotic proteomes include more disordered regions than other proteomes [30], [47], [48] we chose for this purpose three eukaryotic proteomes: human (50104 protein sequences), the fruit fly (*Drosophila melanogaster*, 14455 protein sequences), and the nematode worm (*Caenorhabditis elegans*, 23507 protein sequences) proteomes. For comparison we also considered three bacterial proteomes: *E.coli* (strains O6-K15-H31, 4605 protein sequences), *Lactococcus lactis* (2383 protein sequences), and *Mycobacterium tuberculosis* (ATCC 25177, 3990 protein sequences). The patterns with the largest occurrence in the eukaryotic proteomes are given in Table 3. It should be underlined here that the patterns with low complexity appear in the eukaryotic proteome more often than others. It should be noted also that low complexity regions can additionally include ordered structural proteins or proteins with strong structural propensity, like collagens, coiled-coils or fibrous proteins [12]. Recently, it has been demonstrated that increasing perfect tandem repeats correlates with a stronger tendency to be unstructured [49]. Moreover, a strong association between homorepeats and unstructured regions has been shown elsewhere [50]. Another characteristic of the patterns with low complexity is that they appear in proteins with different functions. For three patterns PPPPPP, QQQQQQ, and HHHHHH we found functional categories in the gene ontology [51] classification (the GO annotation). This was done as follows. We took eukaryotic proteomes from the EBI site (ftp://ftp.ebi.ac.uk/pub/databases/SPproteomes/uniprot/proteomes/). From these proteomes for each protein with the pattern we took the GO molecular function classification (GO:F section). We focus our attention only on molecular functions if there are at least five proteins in human proteome where the pattern occurs.

Molecular functions for the proteins including the PPPPPP pattern: actin binding, calcium ion binding, DNA binding, nucleic acid binding, protein binding, protein serine/threonine kinase activity, receptor activity, Rho GTPase binding, RNA binding, SH3 domain binding, signal transducer activity, transcription coactivator activity, transcription factor activity, tropomyosin binding, voltage-gated potassium channel activity, and zinc ion binding.

Molecular functions for the proteins including the QQQQQQ pattern: DNA binding, nucleic acid binding, protein binding, RNA binding, transcription factor activity, and zinc ion binding.

Molecular functions for the proteins including the HHHHHH pattern: protein binding, transcription coactivator activity, transcription factor activity, and zinc ion binding. It should be noted that poly H fragments are artificial parts of proteins in PDB which have been added for better purification of proteins, but in the eukaryotic proteomes (HHHHHH is absent in the bacterial proteomes at all) such a repeat is likely to have a biological function. It should be added, that poly H and poly Q patterns occur in the fruit fly proteome more often than in the human proteome (see Table 3).

We have found a very interesting example of protein Serine arginine-rich pre-mRNA splicing factor SR-A1 (including 1312 amino acid residues) with the RNA binding molecular function where there is compositional bias to regions with low complexity: Pro-rich, Ser-rich, Glu-rich, Arg-rich, and Lys-rich (the protein includes six low complexity patterns: EEEEEE, PPPPPP, RRRRRR, SSSSSS, APAPAP, DRDRDR). Another interesting example with the same situation is AT-rich interactive domain-containing protein 1A (including 2285 amino acid residues) with the DNA and protein binding molecular function (the protein includes six low complexity patterns: AAAAAA, EEEEEE, GGGGGG, PPPPPP, QQQQQQ, SSSSSS).

As expected, the number of occurrences of patterns in the bacterial proteomes is considerably less than in the eukaryotic proteomes. The appearance of the only pattern PPPPPP more than 10 times (11 occurrences) we observed in the *M. tuberculosis* proteome.

It should be underlined here that expansion of homorepeats is a molecular cause of at least 18 human neurological diseases [49]. Therefore, studying the functional role of the obtained patterns, especially homorepeats in the human proteome is one of important biology tasks.

Combining motif discovery and disorder protein segment identification in PDB allows us to create the library of the disordered patterns. At present the library includes 109 disordered patterns. Such an approach is new and promising for further studying and understanding the functional role of the obtained patterns in different proteomes.

## Supporting Information

Dataset S1 The list of patterns and their properties.
(0.05 MB XLS)

Dataset S2 The list of correlated patterns.
(0.14 MB XLS)
